# 

**Published:** 1906-06-15

**Authors:** 


### ANNOUNCEMENTS.

DR. J. JONES.—The many friends of Dr. Jones, of Jackson, Ohio, will be glad to learn that he has so far recovered from his recent long illness that he hopes soon to be serving his patients with his former effectiveness. He graduated from the Ohio College of Dental Surgery in 1874, and has been in practice continuously since then, we think, at Jackson. He is well known all through Southern Ohio, as he has always taken an active interest in dental matters, and has enjoyed the confidence of a large clientelle.

INTERSTATE DENTAL FRATERNITY.—The date of this meeting is changed to September 17th, 1906.

R. M. SANGER, *Secretary.*

—✦—

OFFICERS ST. LOUIS SOCIETY OF DENTAL SCIENCE for the ensuing year are: President, Adam Flickinger; vice-president, Emma Eams Chase; Secretary, Geo. H. Westhoff; curator, R. Summa; Censors, E. P. Dameron, D. O. M. Le Cron, F. S. McKay.

—✦—

C. KINGSLEY FIELD.—Dr. Kingsley Field, who attended the University of Michigan for his freshman and junior years of professional education and graduated at Howard, in 1904, has located in London, England, for practice with his father, Dr. George Field, at 64 Addison Road, Kensington, W.

—✦—

CHARLES H. OAKMAN, M. D.—Dr. Charles H. Oakman, of Detroit, graduated from the Detroit College of Medicine, May 17th. Dr. Oakman is a member of the Michigan State Board of examiners, and has a large dental practice in Detroit. He has done considerable work in oral surgery, and expects to specialize in this subject more than he has, and has taken the medical degree to better qualify him for the work.

—✦—

WILLIAM G. LAW, D. D. S.—Dr. Law has severed his connection with Drs. Jenkins and McBride, at Dresden, Germany, and located in den Zelten, 18a, Berlin, Germany, for the exclusive practice of orthodontia

COMMENCEMENT OF OHIO COLLEGE OF DENTAL SURGERY. —The 60th annual commencement of this school was held at the Odion, May 10th, 1906. Twenty-nine seniors were graduated. The address for the faculty was delivered by Rev. Geo. A. Thayer, and for the Class by Jno. C. McKee.

—✦—

DR. L. P. BETHEL.—Dr. Bethel, who for several years has been Dean of the Ohio Medical University, Dental Department, has resigned his chair and deanship, and will devote his time to the exclusive practice of Orthodontia in Columbus, Ohio. Dr. H. M. Seman, will be the new dean.

—✦—

DENTAL DEPARTMENT, DETROIT COLLEGE OF MEDICINE. —The commencement of exercises of this school were held June 7th, 1906, at the Detroit Opera House. Twenty-one seniors were graduated. Dr. H. W. Heasley, delivered the address for the faculty, and O. L. Brooks for the graduating class.

—✦—

THE ILLINOIS STATE DENTAL SOCIETY.—This society now has a membership of more than 1,300, and is therefore the largest state society in the country. At the recent meeting at Springfield there were in attendance about 500 and the sessions were full of interest from beginning to end. A feature of the meeting was a complimentary banquet to Senator A. C. Clark, through whose influence the dental bill was passed a year ago. Governor Deneen was present and made a most impressive speech, and the event was one long to be remembered by those present. Senator Clark was made the recipient of a beautiful souvenir volume containing testimonials from all the organized dental societies and colleges in the state—a memento that any legislator might well be proud of. In this event the dental profession of Illinois has set a good example in showing public appreciation of worthy effort put forth in securing proper legislation for the protection of the people of the state.

Another presentation was that of a mahogany library table and a Persian rug to Dr. A. D. Black in recognition of his services as chairman of the re-organization committee.

It was a love feast all round and the meeting of 1906 will long be remembered by those in attendance. The officers of the society for the coming year are as follows:

President, Elgin MaWhinney, Chicago; vice-president, T. P. Donelan, Springfield; secretary, Arthor D. Black, Chicago; treasurer, Chas. P. Pruyn, Chicago; librarian, J. T. Cummins, Metropolis; program committee, C. L. Snyder, Freeport; clinic committee, C. N. Thompson, Chicago; chairman local committee, Henry L. Whipple, Quincy.

Next meeting Quincy, May 7, 8, 9 and 10, 1907.

—✦—

RECIPROCITY IN IOWA.—The following amendment to the dental law of Iowa has been passed by the Thirty-first General Assembly.

*Be it enacted by the General Assembly of the State of Iowa:*

SECTION 1. The Board of Dental Examiners may, without examination, issue license to practice to any dentist who shall have been in legal practice in some other State or Territory for a period of at least five years, upon the certificate of the Board of Dental Examiners or a like board of the State or Territory in which such dentist was a practitioner certifying his competency and that he is of good moral character and upon payment of twenty-five dollars ($25.00). Provided, however, that the State from which any practitioner may come shall have, and maintain equal standards of laws regulating the practice of dentistry and recognize exchange certificates issued by the Board of Dental Examiners of the State of Iowa.

"Scrap Pile for San Francisco Sufferers.—Editor Dental Register: Under the heading of the "Scrap Pile" I desire to make a suggestion regarding a fund for San Francisco sufferers. From my experience during the past few weeks, the task of acquiring a sum sufficiently large to materially assist those in need is a difficult one, and the "Scrap Pile" idea envolved from the ease with which a goodly sum was collected in Kansas City a year ago for an event of minor importance by simply requesting all asked to donate a a portion of their gold scraps. Several hundred pennyweight soon accumulated.

Now let every dentist, regardless of the fact whether or no he has paid into other funds, box up a portion of his gold scraps and send them to any dental journal, or to Dr. J. D. Patterson, of Kansas City, Mo., treasurer of National Relief Committee, Keith & Perry Building, and further assist those in distress.

Yours,

J. P. Root, Editor *Kansas City Journal.*

—✦—

Michigan State Dental Association.—The 50th anniversary of the organization of this Society will be held at Detroit, July 9, 10 and 11, and great efforts are being made to secure a large attendance. There will be special exercises in commemoration of the organization, and a review of the work accomplished by the society during its career. An excellent program of up-to-date interest in both the clinical and literary features is being prepared, and the Detroit Dentists will try to keep their reputation for hospitality. It will be an excellent meeting, the time will be favorable for a trip to this great summer resort, and every one who can attend will be pleased and profited.

